# Rapid antiretroviral therapy initiation following rollout of point-of-care early infant diagnosis testing, Uganda, 2018–2021

**DOI:** 10.1186/s12981-024-00613-8

**Published:** 2024-05-15

**Authors:** Stella M. Migamba, Tamara Nsubuga Nyombi, Edirisa Juniour Nsubuga, Andrew Kwiringira, Augustina Delaney, Steven Ndugwa Kabwama, Mary Nakafeero, Benon Kwesiga, Daniel Kadobera, Phoebe Monalisa-Mayambala, Lilian Bulage, Alex Riolexus Ario, Julie R. Harris

**Affiliations:** 1 Uganda Public Health Fellowship Program, Uganda National Institute of Public Health, Kampala, Uganda; 2 Division of Global HIV and Tuberculosis, U.S. Centers for Disease Control and Prevention, Kampala, Uganda; 3 Division of Global HIV and Tuberculosis, U.S. Centers for Disease Control and Prevention, Atlanta, Georgia; 4 Makerere University School of Public Health, Kampala, Uganda; 5 Division of Global Health Protection, U.S. Centers for Disease Control and Prevention, Kampala, Uganda; 6 United States Agency for International Development (USAID), Kampala, Uganda

**Keywords:** Point-of-care, Early infant diagnosis, Antiretroviral therapy, HIV-exposed, Uganda

## Abstract

**Background:**

Uganda Ministry of Health (MOH) recommends a first HIV DNA-PCR test at 4–6 weeks for early infant diagnosis (EID) of HIV-exposed infants (HEI) and immediate return of results. WHO recommends initiating antiretroviral therapy (ART) ≤ 7 days from HIV diagnosis. In 2019, MOH introduced point-of-care (POC) whole-blood EID testing in 33 health facilities and scaled up to 130 facilities in 2020. We assessed results turnaround time and ART linkage pre-POC and during POC testing.

**Methods:**

We evaluated EID register data for HEI at 10 health facilities with POC and EID testing volume of ≥ 12 infants/month from 2018 to 2021. We abstracted data for 12 months before and after POC testing rollout and compared time to sample collection, results receipt, and ART initiation between periods using medians, Wilcoxon, and log-rank tests.

**Results:**

Data for 4.004 HEI were abstracted, of which 1.685 (42%) were from the pre-POC period and 2.319 (58%) were from the period during POC; 3.773 (94%) had a first EID test (pre-POC: 1.649 [44%]; during POC: 2.124 [56%]). Median age at sample collection was 44 (IQR 38–51) days pre-POC and 42 (IQR 33–50) days during POC (p < 0.001). Among 3.773 HEI tested, 3.678 (97%) had test results. HIV-positive infants’ (n = 69) median age at sample collection was 94 (IQR 43–124) days pre-POC and 125 (IQR 74–206) days during POC (p = 0.04). HIV positivity rate was 1.6% (27/1.617) pre-POC and 2.0% (42/2.061) during POC (p = 0.43). For all infants, median days from sample collection to results receipt by infants’ caregivers was 28 (IQR 14–52) pre-POC and 1 (IQR 0–25) during POC (p < 0.001); among HIV-positive infants, median days were 23 (IQR 7–30) pre-POC and 0 (0–3) during POC (p < 0.001). Pre-POC, 4% (1/23) HIV-positive infants started ART on the sample collection day compared to 33% (12/37) during POC (p < 0.001); ART linkage ≤ 7 days from HIV diagnosis was 74% (17/23) pre-POC and 95% (35/37) during POC (p < 0.001).

**Conclusion:**

POC testing improved EID results turnaround time and ART initiation for HIV-positive infants. While POC testing expansion could further improve ART linkage and loss to follow-up, there is need to explore barriers around same-day ART initiation for infants receiving POC testing.

## Background

In 2015, the ‘Start Free, Stay Free, AIDS Free’ global framework was launched to fast-track the HIV response for children, adolescents, and young women by 2020 [1]. The strategy of ‘AIDS free’ is the provision of HIV diagnosis, treatment and care to children and adolescents living with HIV [1]. Global partners selected 23 focus countries with 21 of them, including Uganda, located in sub-Saharan Africa. The number of children aged 0–14 years who acquired HIV in the 21 focus countries in 2021 was approximately 110,000, much higher than the framework’s global target of reducing new HIV infections among children to less than 20,000 annually by 2020 [1].

Early infant diagnosis (EID) involves the testing of HIV-exposed infants before they reach 2 months of age to establish timely diagnosis of HIV and access to life-saving treatment [2]. The Joint United Nations Programme on HIV/AIDS (UNAIDS) target for eliminating vertical HIV transmission from mothers to their infants is to ensure that 95% of HIV-exposed infants (HEI) receive a virologic test and parents are provided the results by 2 months of age. This is part of the 2025 AIDS targets [3]. However, in 2020, only 68% of HEI globally were tested by 2 months of age [4].

In Uganda, the HIV vertical transmission rate reduced from 12.1% in 2015 to 6.8% in 2021 [5]. Concurrently, the EID coverage (proportion of HEI tested by 2 months of age) improved from 44.5% in 2015 to 74.5% in 2021 [5]. The Uganda Ministry of Health (MOH) standards for EID testing, adopted from the WHO 2016 guidelines, recommend that infants born to women living with HIV have their first EID test done at 4–6 weeks of age or as soon as the infant is identified thereafter as being born to an HIV-positive mother [6]. Conventional EID of HIV involves collecting dried blood spots (DBS) from HEI at health facilities and conducting deoxyribonucleic acid polymerase chain reaction (DNA PCR) tests on these samples at a specialized reference laboratory. Infants aged < 18 months suspected to have HIV or with unknown exposure status should be screened for exposure, tested if exposed, and immediately linked to anti-retroviral therapy (ART) if HIV-positive [7]. WHO recommends rapid ART initiation (within 7 days, and on the same day if ready) for people diagnosed with HIV including children [8]. Providing rapid results reduces loss to follow-up and mortality in infants with HIV infection [7].In 2019, 75,000 HEI in Uganda who were below 18 months of age received a first DNA PCR test. However, only 71% of these received the test within 2 months of birth [9]. To facilitate more rapid turnaround time for HIV test results in infants, in 2019 the Uganda MOH rolled out whole-blood point-of-care (POC) EID testing in 33 health facilities providing prevention of mother-to-child transmission (PMTCT) of HIV and EID service delivery across the country; in 2020, it was scaled up to 130 health facilities [10]. In Uganda, two WHO prequalified POC platforms are used- Cepheid GeneXpert and Abbott m-Pima q HIV-1/2 Detect [11].

Studies in Malawi and Mozambique after POC rollout yielded > 98% result receipt by caregivers for infants undergoing POC testing, and > 70% started on ART on the same day they received their results [12, 13]. However, the impact of POC testing on EID turnaround time and linkage to ART among HIV-positive infants in Uganda is unknown. We assessed turnaround time and ART linkage pre-POC and during POC testing with the aim of generating evidence to improve EID testing timeliness and coverage and enabling faster linkage of HEI to ART.

## Methods

### Study setting

We collected data for the period of April 2018–September 2021 at 10 health facilities with POC EID testing. Health facilities in Uganda are classified into seven levels. In ascending order, these are: clinic (community-based preventive and promotive health services), Health Centre Two (HC II), Health Centre Three (HC III), Health Centre Four (HC IV), general hospital, regional referral hospital (RRH), and national referral hospital (NRH) [14]. We collected data at three RRH (Fort Portal RRH, Mubende RRH, and Kawempe RRH), four general hospitals (Kiboga Hospital, Lyantonde Hospital, Mityana Hospital, and Kyenjojo Hospital), and 3 HC IVs (Kyegegwa HC IV, Mpigi HC IV, and Sembabule HC IV). With the exception of Fort Portal RRH which had Cepheid GeneXpert, the rest of the nine health facilities used Abbott m-Pima q HIV-1/2 Detect. Kiboga hospital also used Cepheid GeneXpert. The study health facilities were selected because they had POC testing introduced, reported the highest numbers of HEI tested for HIV in 2020 in their regions, and had a minimum EID testing volume of 12 infants per month according to the District Health Information System version 2 (DHIS2), a national electronic health database. The 10 sites were selected from the initial pool of 33 pioneer sites being an opportunity for the study to leverage the experience gained from early implementation efforts.

Socioeconomically, agriculture is the main source of income in 53% of the households in Uganda [15].

### Study design and data source

We conducted a retrospective evaluation of data for HEI at the 10 health facilities before and after the implementation of POC EID testing. At each facility, we abstracted data from EID registers for 12 months following the rollout of POC testing (during POC) at the facility. Since POC testing was introduced at health facilities at different times, the POC period ranged from April 2019 to September 2021. For comparison, we also abstracted data for 12 months before POC rollout (pre-POC period) when centralized testing at a reference laboratory was the standard of care. The pre-POC period ranged from April 2018 to September 2020. According to national guidelines, two HIV DNA PCR tests are conducted for HEI. The first test should be done at 4–6 weeks. HEI with a negative first HIV DNA PCR test should be retested using the same test 6 weeks after cessation of breastfeeding. Those with a negative second HIV DNA PCR should receive a final rapid HIV antibody test at 18 months [16]. The study utilized results for the first DNA PCR test.

### EID procedures

Under POC testing, the health facilities had different set-ups based on size and type of machine. For example, sites with Cepheid GeneXpert machines had them placed in the laboratory which is a separate room or site, while sites with Abbott m-Pima q HIV-1/2 Detect machines had them at mother-baby care points (MBCP) and some in the laboratories. Generally, either the sample was drawn at the clinic and sent to the laboratory or the client was sent to the laboratory. The sample was processed at the POC (either MBCP or laboratory) where it was run through the machine; this could take up to an hour. Results were printed, returned to the clinic, and given to clients within the same day to the greatest extent possible.

During Pre-POC period, DBS samples were sent to the Central Public Health Laboratory (CPHL), the central reference laboratory via the hub system. Samples were collected at health facilities, delivered by a laboratory hub rider from the health facilities to the laboratory hub. A CPHL driver then picked the samples twice a week and delivered them to CPHL where they were sorted, coded and then tested. It took 3–7 days to test samples. Results were uploaded on the EID results dashboard, the hub downloaded these results, and the hub rider delivered them to the health facilities. At health facilities, results were recorded and given to the caregivers at follow up visits which could be between 2 weeks and 3 months.

### Study variables and data collection

We used Kobo Collect application to program a questionnaire on tablets. The questionnaire included infant’s identification number, sex, date of birth, date of registration at the facility, date of collection of first PCR test, dates results received, dates results given to caregiver, ART enrollment status and date, and final EID outcome (discharged negative, referred for ART, lost, died negative, died positive). We used the date of registration of the infants to determine whether they were registered for HIV testing pre-POC or during POC. HIV DNA PCR result turnaround times were defined as the number of days from sample collection to return of results to the clinic, or results return to the caregiver. HIV diagnosis date was defined as the time when HIV test results were received at the clinic. For turnaround time from sample collection to results receipt at the clinic, we used dates that results were received at the clinic or date of last clinic visit (date of last follow-up) for censored observations. Censored observations were those for which survival times were unknown because they had no date of results returned. For turnaround time from sample collection to results receipt by caregiver, we used dates of caregiver results receipt.

A sub-group analysis was conducted among infants who tested positive for HIV to assess turnaround times and the effect of POC testing on time to ART initiation. The primary outcome in this study was time to ART initiation. Time to ART initiation was defined as the number of days between dates of sample collection and initiation on ART; same-day ART initiation was defined as starting ART on the same day of sample collection. Positivity rate was defined as the proportion of infants that tested HIV positive out of the number tested who had valid results. The secondary study outcome was time to first HIV DNA PCR sample collection from birth. When calculating proportion of infants with HIV test results, only HEI who had date of result returned to the clinic were included in the analysis.

### Data management

Trained research assistants working in the EID clinics at health facilities completed the questionnaire. Data were sent from the tablet computers to the Kobo Collect server each day. We analyzed the data in Stata version 14. Duplicate entries were removed using the exposed infant identification number and health facility name. HEI missing dates for a step in the care cascade were excluded in the analysis for that particular step.

### Data analysis

We calculated summary statistics for all variables. Categorical variables were presented as frequencies and proportions and continuous variables were described using medians and interquartile ranges. We compared time to sample collection, results receipt at the clinic and by the caregiver, and ART initiation between pre-POC and POC periods using the Wilcoxon rank-sum test and Kaplan Meier curves. The log-rank test was used to test for differences in time to ART initiation between the pre-POC period and the POC period as displayed in Kaplan–Meier curves.

## Results

We abstracted data for 4.004 HIV-exposed infants, 40% of which were from general hospitals or regional referral hospitals. Fifty one percent of HEI were male. The overall median time from birth to sample collection was 43 days (IQR 34–51). The median age at sample collection was slightly older for all infants pre-POC than during POC (44 vs 42 days, p < 0.001). Among 69 infants infected with HIV, samples were collected at a later age during POC than the pre-POC period (median 125 vs 94 days, p = 0.04) (Table 1).

**Table 1:** Socio-demographic and testing characteristics of HIV exposed infants pre- and post-point of care testing initiation at ten health facilities, Uganda, April 2018–September 2021

Characteristic	Pre-POC		POC		p-value
	n	%	n	%	
Health facility level (n = 4.004)					
Health Centre IV	347	21	416	18	0.08
General Hospital	683	40	1.015	44	
Regional referral hospital	655	39	888	38	
Sex^a^ (n = 3.812)					
Male	853	51	1.041	48	0.07
Female	807	49	1.111	52	
Age at sample collection in days					
All infants (n = 3.773)					
< 60	1,364	83	1,765	83	< 0.001
60–180	255	15	261	12	
181–365	26	1.6	80	4	
> 365	4	0.2	18	1	
Median (IQR)	44	(38–51)	42	(33–50)	< 0.001
HIV-positive infants (n = 69)					
< 60	9	32	9	22	0.24
60–180	15	57	20	46	
181–365	2	7	9	22	
> 365	1	4	4	8	
Median (IQR)	94	(43–124)	125	(74–206)	0.04

^a^192 infants did not have their sex recorded, 25 pre-POC and 167 POC

### Testing characteristics of the HIV-exposed infants

**Fig. 1:**
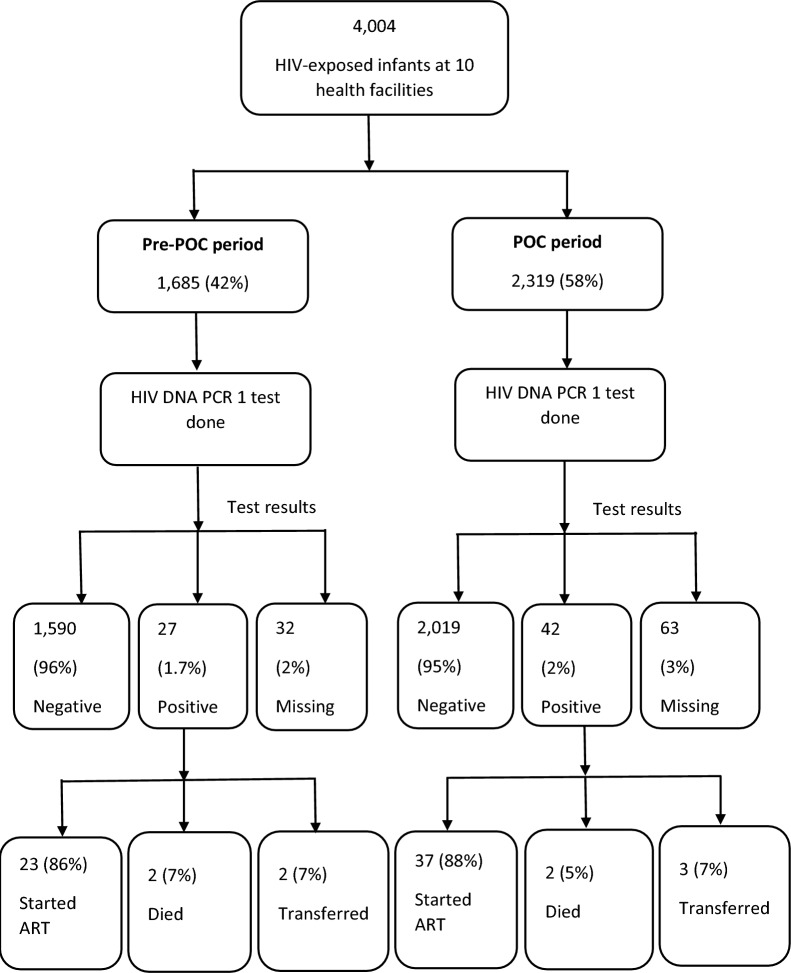
Flow chart for HIV-exposed infants’ cohorts pre- and post-point-of-care testing initiation at ten health facilities, Uganda, April 2018─September 2021

Of 4.004 HEI, 1.685 (42%) were from the pre-POC period and 2,319 (58%) from the POC period. Of these, 94% (3.773/4,004) had a first HIV DNA PCR test done, including 44% (1.649/3.773) pre-POC and 56% (2.124/3.773) during POC (p < 0.001). Of those tested, 97% (3.678/3.773) had results. Sixty-nine (1.9%) infants tested positive; the proportion positive was similar in the pre-POC (1.7%) and during POC (2.0%) periods (p = 0.43). Sixty (87%) infants infected with HIV in this study were initiated on ART (Fig. 1). Same-day receipt of results at the clinic was more frequent during POC than pre-POC (46 vs 10%, p < 0.001), as was the same-day receipt of results by the caregiver (40 vs 6%, p < 0.001). During POC period, 95% (35/37) infants infected with HIV were initiated on ART within seven days of test results at the clinic compared to 74% (17/23) in the pre-POC period (p < 0.001); 54% (20/37) of infants started ART immediately following diagnosis (on the same day as test result at clinic) during POC compared to 17% (4/23) pre-POC (p < 0.001) (Table 2).The age of HEI at the time of receipt of first HIV DNA PCR test results by their caregivers decreased from 96 days pre-POC to 50 days during POC (p < 0.001). Median time from sample collection to results receipt by the caregiver decreased from 28 days pre-POC to 1 day during POC (p < 0.001), and median time from sample collection to ART initiation decreased from 24 days pre-POC to 1 day during POC (p < 0.001) (Table 3).

**Table 2:** Time (in days) between steps in the EID care cascade for HIV-exposed infants pre- and post-point-of-care testing initiation at ten health facilities, Uganda, April 2018–September 2021

Turnaround time	Pre-POC		POC		P value
	n	%	n	%	
Time from sample collection to result receipt at clinic (days)^a^ (n = 3.627)					
Same day (0)	164	10	928	46	< 0.001
1–7	191	12	408	20	
8–28	790	50	452	22	
29–60	368	23	179	9	
> 60	85	5	62	3	
Time from sample collection to result receipt by care giver (days)^b^ (n = 3.521)					
Same day (0)	88	6	780	40	< 0.001
1–7	193	12	430	22	
8–28	503	32	336	17	
29–60	497	32	255	13	
> 60	287	18	152	8	
Time from sample collection to ART initiation (days) (n = 60)					
Same day (0)	1	4	12	33	< 0.001
1–7	2	9	13	35	
8–28	11	48	9	24	
29–60	7	30	3	8	
> 60	2	9	0	0	
Time from result receipt at clinic to ART initiation (days) (n = 60)					
Same day (0)	4	17	20	54	< 0.001
1–7	13	57	15	40	
8–28	3	13	1	3	
29–60	3	13	1	3	
Time from result receipt by caregiver to ART initiation (days) (n = 60)					
Same day (0)	19	83	26	70	0.39
1–7	3	13	9	24	
> 7	1	4	2	5	

^a^146 infants did not have results returned to clinic, 51 pre-POC and 95 during POC

^b^251 infants did not have results returned to their care givers, 81 pre-POC and 170 during POC

**Table 3:** Turnaround times (days) and age of HEI at different steps in the EID care cascade pre- and post-point-of-care testing initiation at ten health facilities, Uganda, April 2018–September 2021

	n	Median (IQR) days		p value
		Pre-POC	POC	
All HIV exposed infants				
Age at result receipt by caregiver	3.521	96 (75–141)	50 (38–89)	< 0.001
Sample collection to results receipt at clinic	3.627	20 (10–30)	1 (0–17)	< 0.001
Sample collection to results receipt by caregiver	3.521	28 (14–52)	1 (0–25)	< 0.001
HIV positive infants				
Age at result receipt by caregiver	69	106 (74–164)	127 (75–206)	0.47
Age at ART initiation	60	118 (71–175)	138 (77–218)	0.56
Sample collection to results receipt at clinic	69	18 (6–29)	0 (0–3)	< 0.001
Sample collection to results receipt by caregiver	69	23 (7–30)	0 (0–3)	< 0.001
Sample collection to ART initiation	60	24 (12–33)	1 (0–12)	< 0.001

Both pre-POC and during POC, HEI who tested HIV-positive (n = 69) were older at sample collection than those who tested HIV-negative (n = 3.609) (100 days vs 43 days, p < 0.0001). HIV positivity rate increased with the age at which infants were tested (Table 4).

**Table 4:** Positivity rate by age at first HIV DNA PCR test among HEI pre- and post-point-of-care testing initiation at ten health facilities, Uganda, April 2018–September 2021

Age at HIV testing (days)	Pre-POC	POC
	n/N (%)	n/N (%)
< 30	2/159 (1.3)	1/270 (0.4)
31–60	7/1.213 (0.6)	8/1.499 (0.5)
61–90	4/145 (2.8)	7/150 (5.0)
91–120	7/59 (12)	4/52 (7.7)
> 120	7/73 (10)	22/153 (14)
Total	27/1.649 (1.6)	42/2.124 (2.0)

**Fig. 2:**
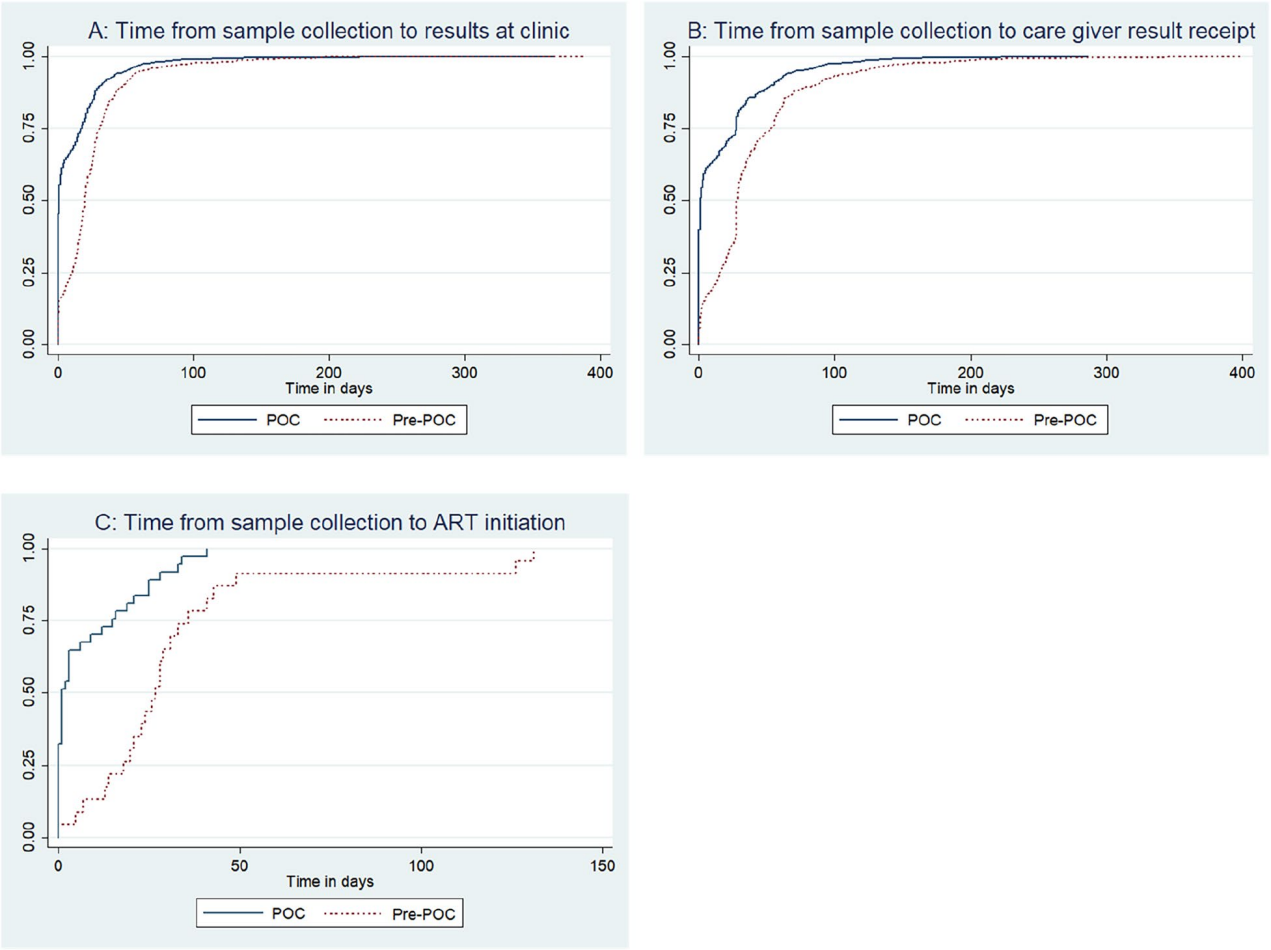
Kaplan Meier curves showing turnaround times from sample collection to clinic receipt of results (**A**), caregiver receipt of results (**B**), and ART initiation (**C**) for HEI at ten health facilities, Uganda, April 2018─September 2021

The time from sample collection to results receipt at the clinic, from sample collection to results receipt by caregiver, and from sample collection to ART initiation for HIV positive infants were shorter during POC than pre-POC (log-rank p < 0.001 for all comparisons) (Fig. 2).

## Discussion

In this evaluation, EID POC testing reduced turnaround times from sample collection to results return to the clinic and caregiver, and improved linkage to ART. This is consistent with previous studies in other African countries [12, 13, 17]. Although 83% of infants received their first HIV DNA PCR test within 2 months of age, this still fell short of the 95% target, demonstrating existing gaps in EID testing that need to be addressed [3]. During both the pre-POC and POC testing periods, all infants infected with HIV who did not die and were not transferred were linked to ART, but POC testing reduced the time to diagnosis of HIV and time to ART initiation.

The proportion of HIV test results for HEI returned to the caregiver on the same day of sample collection during POC was lower in this study (40%) compared to studies elsewhere. Data from six countries in Africa showed that POC testing resulted in 72% of infants receiving their results on the same day of sample collection [14], while studies in Malawi and Mozambique resulted in 99.5 and 98% of results being received by caregivers on the same day as sample collection during POC testing [12, 13]. Anecdotally, six of the ten health facilities reported periods of stockout of cartridges used in POC machines during which the facilities reverted to conventional EID testing. This could have contributed to the longer time from sample collection to caregiver receipt of results in this study. In addition, the COVID-19 pandemic disrupted essential health services in Uganda, which led to delayed delivery of HIV/AIDS care commodities [18, 19]. During this period, stockouts of critical reagents and real-time PCR diagnostics, including GeneXpert cartridges used for POC testing, were documented at some health facilities due to repurposing to support the COVID-19 response [20]. Previously, sub-optimal use of POC testing instruments and instrument downtime were identified as challenges affecting elimination of mother-to-child transmission of HIV (eMTCT) [10, 21]. These factors may at least partially explain the delayed turnaround times identified in our study. Strengthened clinical and laboratory management systems could support HEI testing, same-day results turnaround, and ultimately retention among HEI tested using POC tests [22].

Despite these challenges, our study demonstrated a reduction in median time from sample collection to results return to the caregiver between pre-POC and during POC periods (28 versus 1 day). This is similar to observations in studies in several countries where POC testing achieved faster turnaround times, with median days from sample collection to results receipt by caregiver ranging from 35 to 56 median days under conventional EID testing to 0 or < 1 day under POC testing [13, 17, 23].

The median turnaround time from sample collection to ART initiation reduced from 24 days pre-POC to 1 day during POC for infants testing positive for HIV. Other studies also demonstrated reduced median turnaround time from sample collection to ART initiation with POC [12, 13, 17, 23]. Additionally, all infants in our study who tested HIV-positive and did not die or were not transferred were initiated on ART within 60 days after sample collection during POC, a modest increase from the observed 91% in the pre-POC period. The high proportion of infants infected who tested HIV-positive and were promptly initiated on ART in this study suggests that the low ART coverage of 60% among children aged 0–9 years in 2021 [10] may be related to poor case-finding among older children, rather than infants. If HEI are identified at high rates, then the very high rates of linkage will eventually improve overall ART coverage in Uganda.

Our findings of 94% of HEI having a first DNA PCR test done and 83% of them having received testing within 2 months of age are slightly higher than the 88% of HEI that had an EID test and 74% who had their first DNA PCR within 2 months of age reported in the National Annual Joint AIDS Review in 2021 [10]. The differences may be related to the different and much smaller population in our study than the population used for the national-level data, as well as the slightly different time periods during which the evaluations were conducted.

The first HIV DNA PCR sample for HEI is meant to be conducted within the first 60 days of life. Due to the introduction of the ‘EID Systems Strengthening’ model in Uganda in 2011, which aimed at improving testing, linkage, and retention of HIV-exposed and infected infants, the mean age at first HIV DNA PCR test had already reduced from 7 months in 2011 to 4.2 months in 2014 [24]. In our study, the median overall age at testing was 1.4 months. Notably, all infants in this study who tested positive for HIV, both pre-POC and during POC, had their first HIV DNA PCR sample collected at a higher median age than those who tested negative (100 vs 43 days). This could be due to the delay in identifying HEI (who later test positive) which in turn delays routine care activities such as ART and co-trimoxazole prophylaxis and follow up, which reduce chances of HEI acquiring HIV [25].

It also reflects the longer time period of exposure among infants who tested HIV-positive than those who tested HIV-negative. Evidence from other studies also shows that infants who test HIV-positive present for testing later than those who test HIV-negative. In Uganda, Kiyaga et al*.* [26] observed that among HEI whose samples were sent to the Central Public Health Laboratory for routine diagnosis, infants who tested HIV-positive were 1.5 months older than those who tested HIV negative. Similarly, in Malawi and Mozambique, HEI whose samples tested positive for HIV presented for testing at older ages than those whose samples were negative [12, 13]. Furthermore, a study in Nigeria revealed that the odds of an HIV-negative result increased with earlier age at testing starting at 6 weeks to beyond 20 weeks [27]. In Uganda, lockdown movement restrictions during the COVID-19 pandemic and the fear of contracting COVID-19 from health facilities disrupted access to HIV/AIDS care services and may have contributed to HEI caregivers not being able to readily access HIV/AIDS care and treatment services [19, 28]. Reduced HIV/AIDS case-finding was also reported in 2020 [29]. These data highlight the importance of earlier identification and testing of HEI from all entry points at health facilities, to reduce opportunities for infection. There is also a need to understand causes of late EID testing, focusing on mother-baby pairs at high risk for HIV transmission to retain them in care until the final outcome status is determined.

## Limitations

This study used secondary data and as such, some data points were missing due to lack of documentation and poor record keeping at many health facilities. Additionally, some infants could have been tested elsewhere and linked to treatment at one of the study facilities leading to longer turnaround times. Furthermore, some health facilities reported stock out of cartridges for POC EID testing and breakdowns of the machines. During such periods, they reverted to conventional EID testing. This might have influenced the turnaround times in this study.

## Conclusion

POC testing improved EID results turnaround times from sample collection to results return to the clinic and sample collection to results return to the caregiver and ART initiation for HIV-positive infants. Later age at testing among infants who turn HIV-positive suggests missed opportunities in identifying and testing HIV-exposed infants. POC testing expansion could further improve ART linkage for HIV positive infants and reduce loss to follow-up. There is need to examine barriers surrounding the POC target of initiating ART on the sample collection day as we aim for eMTCT.

## Data Availability

The data upon which our findings are based belongs to the Uganda Ministry of Health and cannot be shared publicly. However, it can be made available by the corresponding author with permission from the Ministry of Health Uganda, Division of Health Information and Uganda Public Health Fellowship Program.
